# Cancer-related symptoms, mental well-being, and psychological distress in men diagnosed with prostate cancer treated with androgen deprivation therapy

**DOI:** 10.1007/s11136-019-02212-x

**Published:** 2019-05-21

**Authors:** Sarah Wilding, Amy Downing, Penny Wright, Peter Selby, Eila Watson, Richard Wagland, David W. Donnelly, Luke Hounsome, Hugh Butcher, Malcolm Mason, Ann Henry, Anna Gavin, Adam W. Glaser

**Affiliations:** 1 Leeds Institute of Medical Research at St James’s, University of Leeds, Leeds, UK; 2 Leeds Institute for Data Analytics, University of Leeds, Leeds, UK; 3 Department of Midwifery, Community and Public Health, School of Nursing and Midwifery, Oxford Brookes University, Oxford, UK; 4 Faculty of Health Sciences, University of Southampton, Southampton, UK; 5 Northern Ireland Cancer Registry, Queen’s University Belfast, Belfast, UK; 6 National Cancer Registration and Analysis Service, Public Health England, Bristol, UK; 7 Division of Cancer and Genetics, School of Medicine, Cardiff University, Cardiff, UK; 8 Present Address: School of Psychology, University of Leeds, Leeds, UK

**Keywords:** Prostate cancer, Psychological distress, Mental well-being, Androgen deprivation therapy, Cancer-related symptoms, Patient-reported outcome measures

## Abstract

**Purpose:**

There are known associations between treatment of prostate cancer (PCa) involving Androgen Deprivation Therapy (ADT) and psychological and physical side effects. We investigate the associations between cancer-related symptoms, health-related quality of life (HRQL), and poor psychological outcomes in men whose treatment for PCa involved ADT.

**Methods:**

A cross-sectional postal questionnaire was administered to UK men 18–42 months post diagnosis of PCa. Men completed items on functional outcomes using the Expanded Prostate Cancer Index Composite (EPIC-26), EuroQol-5D (EQ-5D), and the European Organisation for Research and Treatment of Cancer (EORTC) Fatigue subscale. Psychological outcomes (mental well-being and psychological distress) were assessed using the Short Warwick–Edinburgh Mental Well-being Scale (SWEMWBS) and the Kessler 6-item scale (K6), respectively. Associations between explanatory variables and psychological outcomes were assessed using stepped logistic regression.

**Results:**

13,097 men treated with ADT completed a questionnaire. A minority of men reported poor mental well-being (15.5%) or severe psychological distress (6.6%). After controlling for sociodemographic and clinical variables, reporting clinically significant fatigue was strongly associated with severe psychological distress (OR 9.92; 95% CI 7.63 to 12.89) and poor well-being (OR 3.86; 95% CI 3.38 to 4.42). All cancer-related symptoms and HRQL variables were associated with both psychological outcomes.

**Conclusions:**

While the majority of men treated with ADT did not report poor psychological outcomes, a small proportion reported severe problems. Clinically significant fatigue was demonstrated as a possible indicator of poor outcomes. Healthcare systems need to have clear protocols in place which specifically and routinely target psychological distress and fatigue.

**Electronic supplementary material:**

The online version of this article (10.1007/s11136-019-02212-x) contains supplementary material, which is available to authorized users.

## Introduction

Prostate cancer (PCa) is the most common cancer in men in the UK [1]. Over the last 40 years, the number of men living with a diagnosis of PCa survival has tripled [2]. With increasing numbers living for long periods with and beyond their diagnosis, understanding and improving the experience of patients post diagnosis and treatment are growing priorities [3]. The National Cancer Research Institute (NCRI) has identified the UK Top 10 Living With and Beyond Cancer research priorities, which includes understanding the short- and long-term psychological impacts of cancer [4, 5]. One group that may be at greater risk of experiencing the negative psychological impact of PCa is the increasingly prevalent group of men, particularly with earlier stage of disease, treated with Androgen Deprivation Therapy (ADT) [6, 7].

There are known associations between ADT and depression, memory difficulties, and fatigue among prostate cancer patients [8–11]. A recent meta-analysis of 18 studies found that risk of depression increased by 41% in men on ADT [12]. The largest study to date of 100,000 men with and without PCa [6] also supported this association; however, when controlling for age, comorbidity, and tumour characteristics, this relationship was no longer significant. Further studies have shown that 19.6% of men on ADT report clinically significant anxiety and that increasing ADT length is associated with poorer quality of life [13], and increased risk of anxiety [14].

Mental well-being is a broad concept, which has been described as the positive aspects of mental health [15]. Non-specific psychological distress occurs in a range of mental health disorders, but is not specific to any one disorder [16]. It therefore provides an indicator of potential mental health problems. Previous studies of PCa have indicated the relationship between the severity of cancer-related symptoms experienced and poorer mental well-being and/or severe psychological distress [17, 18]. However, little work has been done regarding the associations with cancer-related symptoms in patients receiving ADT despite its links to physical (vitality, energy, and fatigue) and sexual dysfunction. There is some suggestion that ADT may indirectly affect risk of psychological distress through an overall reduction in quality of life [19]. The use of Patient-Reported Outcome Measures (PROMs) in large surveys allows clinicians to generate more confident estimates of this kind and therefore allow the planning of appropriate clinical responses.

The Life After Prostate Cancer Diagnosis (LAPCD) study is a UK-wide, population-based, cross-sectional study investigating a range of quality of life (QoL) outcomes in men 18–42 months post diagnosis of PCa. As part of the LAPCD study, Downing et al. [20] recently reported that later-stage PCa was associated with fatigue and sexual dysfunction and this was suggested to be due to receiving ADT. While previous studies have investigated cancer-related symptoms and psychological outcomes in PCa [17], no large-scale study has focused its attention on treatments involving ADT. Qualitative interviews conducted alongside the quantitative LAPCD survey have also supported ADT treatment as being related to changes in mood along with bodily changes, such as mood swings and loss of muscle mass, which were related to distress (Matheson et al. submitted).

Aim: As part of the LAPCD study, we aim to investigate which cancer-related symptoms and health-related quality of life (HRQL) variables are associated with mental well-being and psychological distress in men 18-42 months post diagnosis of PCa who reported receiving ADT.

## Methods

The LAPCD study design has been detailed previously [21]. Men diagnosed with PCa between 18 and 42 months previously were identified through national cancer registration systems in England, Wales, and Northern Ireland, and through hospital activity data in Scotland. These men were sent a postal survey between October 2015 and November 2016. The survey comprised validated PROMs covering generic, cancer, and PCa-specific outcome domains alongside those addressing quality of life, and psychological and social outcomes. The full questionnaire is available in Online Resource 1.

Age was self-reported, and where missing, was supplemented by cancer registration records. Participants also self-reported employment status, ethnicity, legal marital status, sexuality, the presence of other long-term conditions (LTCs) from a list of co-morbidities (e.g. diabetes, heart disease), and carer status. Body Mass Index (BMI) was calculated using self-reported height and weight. An area-based measure of socio-economic deprivation was derived from postcodes using the Index of Multiple Deprivation (IMD) [22–25]. Men were asked to report whether or not in the past they had ever in their lifetime seen a healthcare professional (e.g. GP, psychiatrist, psychologist, counsellor) for problems with emotions or nerves or use of alcohol or drugs (referred to from here on as history of help-seeking for mental health problems/alcohol/drugs). Cancer stage at time of diagnosis was provided by national cancer registries. Treatment received was self-reported. Men were split into those receiving ADT and those not receiving ADT based on whether they had indicated that they had received hormone treatment (alone or in combination with external beam radiotherapy [EBRT], surgery or other systemic treatment; see Online Resource 2). The duration of ADT use was not assessed.

### Cancer-related symptoms

The EuroQol-5D-5L (EQ-5D-5L) includes questions on five dimensions (mobility, self-care, usual activities, pain/discomfort, and anxiety/depression). Each dimension item has five response options (e.g. “I have no problems walking about” up to “I am unable to walk about”). The EQ-5D also includes a rating of self-assessed health (SAH) based on “how good or bad your health is today”, ranked from 0 to 100 where 100 represents best possible health. The proportion of respondents reporting any problem, regardless of severity, in each dimension separately and across four of the five dimensions was computed along with average SAH ratings.

The Expanded Prostate Cancer Index Composite (EPIC-26) is a 26-item measure of function and associated bother over five domains (urinary incontinence, urinary irritation and obstruction, bowel, sexual, and hormonal function) [26]. Each domain includes a question relating to ‘bother’ (“how big a problem has your function been for you during the past 4 weeks?” on a five-point scale: no/very small/small/moderate/big problem). The proportion of respondents reporting moderate or big bother in each domain was computed [20]. Scoring for the EPIC-26 does not provide cut-offs to indicate clinical significance of symptoms.

The European Organisation for Research and Treatment of Cancer (EORTC)-Fatigue subscale includes three items: During the past week (Did you need to rest; have you felt weak; were you tired) scored between ‘not at all’ and ‘very much’, where a high score indicates more problems. A score greater than 39 is considered to indicate clinically significant fatigue [27].

### Psychological outcome measures

The Short Warwick–Edinburgh Mental Well-being Scale (SWEMWBS) [28] is a seven-item measure of mental well-being. Respondents complete items relating to their experiences, thoughts, and feelings ‘over the past 2 weeks’. Scores range from 7 to 35 with higher scores indicating greater well-being. It is suggested that scores ≤ 19.25 indicate poor mental well-being [29].

The Kessler 6 (K6) is a validated measure of psychological distress which asks individuals to report their experiences over the past 30 days and is assessed using a five-point Likert style scale, anchored between 5 (“All of the time”) and 1 (“None of the time”) [30, 31]. Using the Australian K6 scoring system, possible scores range between 6 and 30, where a score of 19–30 indicates severe psychological distress (and possible serious mental illness).

Only the first four dimensions of the EQ-5D and EPIC-26 were included in these analyses due to a lack of independence between the anxiety/depression dimension and hormonal function and both outcome measures.

### Analysis summary

Analyses were limited to men that self-reported receiving ADT either alone or in combination with other treatments.

Missing data were imputed in order to reduce potential bias associated with only including cases with complete data [32, 33]. Multiple imputation with chained equations was utilised [34] based upon all sociodemographic and clinical characteristics and all bother and HRQL items, along with the EORTC-fatigue subscale and both outcomes (severe psychological distress and poor mental well-being). Ten separate imputations were completed [34], with results combined using Rubin’s rules [35]. All respondents were therefore included in analyses. A secondary complete case analysis without multiple imputation was also conducted (Online Resources 3, 4).

The pre-defined cut-offs for SWEMWBS (≤ 19.25) and K6 (> 19) were used as binary outcomes to indicate poor mental well-being and severe psychological distress, respectively.

Stepped-regression analyses were conducted as follows:

1. *Descriptive statistics* Chi-squared analyses were conducted to assess relationships between sociodemographic and clinical background variables and SWEMWBS/K6.
2. *Core model development* Multivariable analyses on each outcome (SWEMWBS and K6) were performed entering all sociodemographic and clinical background variables (Age, LTCs, Employment, Ethnicity, Marital status, Deprivation, Carer status, Mental health help-seeking, BMI, Nation, and Stage as explanatory variables). Those that were significant in this analysis were classified as the ‘core’ models (see Table 2 for the list of variables included in core models). To avoid dropping variables which might be borderline significant following the addition of the variables in step 3, a significance level of 0.25 was used, any variables above this were dropped from the core model.
3. *Final models* Each cancer-related symptom (binary bother for urinary incontinence, urinary irritation, bowel function, sexual function, and fatigue (no clinical fatigue vs. clinical fatigue), continuous EQ-5D SAH, and binary no problems vs. some problems with mobility, self-care, usual activities, and pain/discomfort) was then entered individually into separate multivariable analyses in addition to the ‘core’ models. This was performed on the two outcomes separately. All cancer-related symptoms were a priori selected for inclusion within the final models.

## Results

### Descriptive statistics

In the overall LAPCD sample, 35,823 men responded to the questionnaire (60.8% response rate) of whom 30,114 men reported receiving one of the most common treatment types (Online Resource 2). 13,097 (43.5%) men reported receiving ADT. These men were older (Mean age = 73.49, SD = 7.38) than the men who did not have ADT (Mean = 69.7, SD = 7.92, *p* < .001). In the group treated with ADT, most men (89.3%; N = 11,696) were aged 65 + and 40.9% (N = 5354) reported having two or more other LTCs (Table 1). Table 1 also shows the levels of missing data by each characteristic, prior to multiple imputation.

**Table 1:** Characteristics of men treated with ADT included in analysis before multiple imputation (numbers and percentages)

Characteristics	N	%	Missing (%)
Total	13,097	100.0	
Age group, years			
< 55	89	0.7	3 (0.01%)
55–64	1309	10.0	
65–74	5696	43.5	
75–84	5128	39.2	
85 +	872	6.7	
Number of LTCs			
0	3192	24.4	0
1	4551	34.8	
2	2867	21.9	
3	1413	10.8	
4+	1074	8.2	
Stage			
I/II	5276	46.2	1667 (12.7)
III	3441	30.1	
IV	2713	23.7	
Employment			
Employed	1729	13.6	362 (2.8)
Unemployed	275	2.2	
Retired	10,679	83.9	
Other	52	0.4	
Ethnicity			
White	12,501	98.9	335 (2.6)
Non-white	261	2.1	
Marital/relationship status			
Married/civil partner	10,321	79.6	126 (1.0)
Separated/divorced	882	6.8	
Widowed	1142	8.8	
Single	471	3.6	
Other	155	1.2	
Deprivation			
1 least deprived	3450	27.0	307 (2.3)
2	3471	27.1	
3	2785	21.8	
4	1889	14.8	
5 most deprived	1195	9.3	
Carer status			
No	9536	75.6	486 (3.7)
Yes	3075	24.4	
Mental health help-seeking			
No	10,643	83.4	328 (2.5)
Yes	2126	16.7	
BMI			
Under/healthy weight (0–25)	3398	28.1	1015 (7.7)
Overweight (25–30)	5749	47.6	
Obese (30+)	2935	24.3	
Nation			
England	11,116	84.9	0
Wales	949	7.3	
Scotland	663	5.1	
NI	369	2.8	
Treatment			
Androgen deprivation therapy (ADT) alone	3116	23.8	
EBRT + ADT	7488	57.2	0
Surgery + EBRT & ADT	901	6.9	
Surgery + ADT	581	4.4	
Systemic therapy + ADT (+/EBRT)	1011	7.7	
Mobility			
No problem	7280	56.1	130 (1.0)
Some problems	5687	43.9	
Self-care			
No problem	10,707	82.4	101 (0.8)
Some problems	2289	18.0	
Usual activities			
No problem	6990	53.9	127 (1.0)
Some problems	5980	46.1	
Pain/discomfort			
No problem	6740	52.1	154 (1.2)
Some problems	6203	47.9	
Urinary bother			
No bother	11,193	86.5	160 (1.2)
Moderate/big bother	1744	13.5	
Bowel bother			
No bother	11,499	88.5	107 (0.8)
Moderate/big bother	1491	11.5	
Sexual bother			
No bother	6541	54.7	1130 (8.6)
Moderate/big bother	5426	45.3	
Fatigue			
No fatigue	7353	61.4	1125 (8.6)
Fatigue	4619	38.6	
SAH (mean, SD)	74	18.6	
Mental well-being			
Well-being	10,367	84.5	822 (6.3)
Poor mental well-being	1908	15.5	
Psychological distress			
No/mild distress	11,681	93.4	590 (4.5)
Severe psychological distress	826	6.6	

*LTCs* long-term conditions, *SAH* self-assessed health

Of the men receiving ADT, the majority of men reported receiving both EBRT and ADT (N = 7488; 57.2%), or ADT alone (N = 3116, 23.8%). Smaller proportions of men reported receiving ADT with both surgery and EBRT (N = 901, 6.9%), with surgery (N = 581, 4.4%), and with other systemic treatments (N = 1011, 8.1%; Table 1). Men treated with ADT alone were older (20.6% in men aged 85 + vs. 1.1% of EBRT and ADT) and were more likely to have metastatic disease than men treated with combined ADT and EBRT (43.5% stage IV disease vs. 9.8%; Online Resource 6).

#### Cancer-related symptoms

Just under 40% of the ADT-treated men reported clinically significant fatigue on the EORTC subscale (38.6%, N = 4619). On the EQ-5D, just under half of men reported some problems with pain/discomfort (N = 6203, 47.9%), mobility (N = 5687, 43.9%) and usual activities (N = 5980, 46.1%). The average SAH score for men treated with ADT was 74.0 (SD = 18.6).

On the EPIC-26, just under half of men on ADT reported moderate/big problems in sexual functioning (45.3%, N = 5426). Smaller proportions of the men reported moderate or big problems with urinary (13.5%, N = 1744) or bowel function (11.5%, N = 1491).

#### Psychological outcomes

12,275 (93.5%) men completed the SWEMWBS. 1908 (15.5%) scored below the SWEMWBS cut-off to indicate poor mental well-being. 12,507 completed the K6 (95.4% complete), 826 men (6.6%) scored below the K6 cut-off indicating severe psychological distress (Table 1).

### Sociodemographic and clinical variable analyses (core models; Table 2)

Scoring below the cut-off for well-being was associated with unemployment at the time of survey, having a greater number of LTCs, living in an area of greater deprivation, having previously visited a healthcare professional for mental health-related problems, and marital status (being separated/divorced).

**Table 2:** Univariable and multivariable (core models) associations between sociodemographic and clinical factors and psychological distress and mental well-being

Characteristic	Poor mental well-being		Severe psychological distress	
	Univariable	Multivariable (core model)	Univariable	Multivariable (core model)
	OR (95% CI)		OR (95% CI)	
Age, years				
< 55	1.00	1.00	1.00	1.00
55–64	1.29 (0.74–2.26)	1.16 (0.64–2.12)	0.79 (0.43–1.42)	0.60 (0.30–1.20)
65–74	0.70 (0.40–1.20)	0.86 (0.47–1.57)	0.36 (0.20–0.64)	0.37 (0.18–0.73)
75–84	0.85 (0.49–1.47)	1.10 (0.60–2.04)	0.31 (0.17–0.55)	0.33 (0.16–0.67)
85+	1.25 (0.71–2.19)	1.54 (0.82–2.90)	0.48 (0.26–0.91)	0.53 (0.25–1.13)
Number of LTCs				
0	1.00	1.00	1.00	1.00
1	1.12 (0.97–1.29)	1.07 (0.92–1.23)	1.21 (0.95–1.55)	1.13 (0.88–1.45)
2	1.63 (1.41–1.90)	1.49 (1.28–1.74)	2.12 (1.66–2.70)	1.90 (1.47–2.45)
3	2.25 (1.89–2.66)	2.00 (1.68–2.39)	3.15 (2.43–4.08)	2.74 (2.08–3.60)
4+	3.06 (2.57–3.65)	2.57 (2.13–3.10)	5.93 (4.64–7.58)	4.88 (3.76–6.33)
Employment				
Employed	1.00	1.00	1.00	1.00
Unemployed	5.56 (4.20–7.36)	3.34 (2.47–4.51)	9.10 (6.53–12.68)	4.37 (3.02–6.34)
Retired	1.19 (1.03–1.39)	1.10 (0.92–1.31)	1.29 (1.02–1.63)	1.49 (1.12–1.97)
Other	3.16 (1.74–5.75)	2.61 (1.40–4.87)	2.36 (0.91–6.12)	1.86 (0.67–5.12)
Ethnicity				
White	1.00	–	1.00	–
Non-white	1.23 (0.89–1.71)		1.80 (1.20–2.69)	
Marital status				
Married/civil partner	1.00	1.00	1.00	–
Separated/divorced	1.69 (1.43–201)	1.38 (1.15–1.67)	1.97 (1.57–2.47)	
Widowed	1.56 (1.33–1.83)	1.38 (1.16–1.64)	1.24 (0.98–1.58)	
Single	1.53 (1.19–1.95)	1.26 (0.97–1.63)	1.19 (0.82–1.73)	
Other	1.47 (0.97–2.87)	1.25 (0.81–1.93)	1.66 (0.96–2.86)	
Deprivation quintile				
1 least deprived	1.00	1.00	1.00	1.00
2	1.20 (1.04–1.39)	1.18 (1.01–1.37)	1.24 (0.99–1.55)	1.19 (0.95–1.49)
3	1.25 (1.07–1.45)	1.16 (0.99–1.35)	1.54 (1.24–1.93)	1.39 (1.10–1.75)
4	1.67 (1.42–2.96)	1.45 (1.22–1.71)	2.44 (1.95–3.06)	1.94 (1.53–2.46)
5 most deprived	2.38 (1.98–2.87)	1.87 (1.53–2.28)	3.38 (2.67–4.28)	2.29 (1.77–2.96)
Carer status				
No	1.00	–	1.00	–
Yes	1.07 (0.95–1.20)		1.11 (0.94–1.30)	
Mental health help-seeking				
No	1.00	1.00	1.00	1.00
Yes	2.39 (2.13–2.68)	2.17 (1.92–2.46)	3.81 (3.27–4.43)	3.13 (2.66–3.69)
Stage				
I/II	1.00	1.00	1.00	1.00
III	1.04 (0.92–1.18)	1.06 (0.93–1.21)	1.14 (0.95–1.37)	1.18 (0.97–1.43)
IV	1.21 (1.06–1.38)	1.22 (1.07–1.40)	1.25 (1.04–1.49)	1.26 (1.04–1.53)
BMI				
< 25 under/healthy	1.00	1.00	1.00	1.00
25–30 overweight	0.86 (0.76–0.98)	0.86 (0.75–0.99)	0.95 (0.79–1.15)	0.90 (0.74–1.10)
30+ obese	1.30 (1.14–1.48)	1.16 (1.00–1.33)	1.70 (1.39–2.08)	1.27 (1.02–1.57)
Nation				
England	1.00	–	1.00	1.00
Wales	1.19 (0.99–1.42)		1.36 (1.07–1.73)	1.32 (1.02–1.71)
Scotland	1.25 (1.01–1.54)		1.38 (1.04–1.84)	1.28 (0.95–1.73)
NI	1.08 (0.80–1.45)		1.34 (0.92–1.97)	1.13 (0.74–1.72)

*N* = 13,097

Models based on imputed data

An OR greater than 1 represents greater odds than the first group listed (reference category) of reporting psychological distress or poor mental well-being

A dash (–) indicates that the variable was not significant in the multivariable analysis and was not included in the core model

The distress core model included: Age, Number of LTCs, Employment, Deprivation, having visited a healthcare professional for mental health/alcohol/drug related problems, BMI, nation, and stage at diagnosis. The mental well-being core model included: number of LTCs, Employment, Marital status, Deprivation, having visited a healthcare professional for mental health/alcohol/drug related problems, BMI, and stage at diagnosis

*OR* odds ratio, *CI* confidence interval

Scoring below the cut-off for distress was associated with the same variables as for well-being, with a few exceptions. Nation and age were significant in the distress model but not the well-being model, and marital status was significant in the well-being model but not the distress model (Table 2).

### Cancer-related symptoms (final models; Table 3)

The final multivariable models included all of the significant sociodemographic and clinical variables from the core models. Each of the cancer-related symptoms from the EPIC-26 and HRQL from the EQ-5D along with the EORTC-fatigue binary variable were individually analysed while controlling for the variables in the core model.

**Table 3:** Multivariable associations between cancer-related symptoms, HRQL and psychological distress and mental well-being among prostate cancer survivors who received ADT, after controlling for variables in the core models

Symptoms and HRQL	Poor mental well-being^a^	Severe psychological distress^b^
	OR (95% CI)	OR (95% CI)
Urinary bother		
No bother	1.00	1.00
Moderate/big bother	2.89 (2.54–3.27)^c^	3.69 (3.12–4.38)
Bowel bother		
No bother	1.00	1.00
Moderate/big bother	2.50 (2.20–2.85)	3.68 (3.11–4.34)
Sexual bother		
No bother	1.00	1.00
Moderate/big bother	1.75 (1.57–1.95)	2.71 (2.29–3.22)
Fatigue		
No fatigue	1.00	1.00
Fatigue	3.77 (3.34–4.25)	9.71 (7.73–12.19)
Mobility		
No problems	1.00	1.00
Some problems	2.98 (2.66–3.33)	4.63 (3.82–5.63)
Self-care		
No problems	1.00	1.00
Some problems	3.98 (3.54–4.47)	6.45 (5.41–7.69)
Usual activities		
No problems	1.00	1.00
Some problems	3.49 (3.09–3.94)	6.53 (5.26–8.11)
Pain/discomfort		
No problems	1.00	1.00
Some problems	2.33 (2.09–2.60)	3.74 (3.07–4.53)
SAH	.95 (.95–.96)	.94 (.94–.95)

*N* = 13,097

Models based on imputed data

The ORs were estimated using separate logistic regression models, controlling for the variables in the core models

SAH Self-assessed health. This was a continuous variable scored out of a total of 100 where a greater score indicates better HRQL. An odds ratio of less than 1 for this variable can be interpreted to mean that a decrease in self-reported health was associated with both psychological distress and poor mental well-being (each 1 point reduction in SAH was associated with odds of 1.05 for reporting psychological distress/poor well-being)

*OR* odds ratio, *CI* confidence interval

^a^The mental well-being core model included: number of LTCs, Employment, Marital status, Deprivation, having visited a healthcare professional for mental health/alcohol/drug-related problems, BMI, and stage at diagnosis

^b^The distress core model included: Age, Number of LTCs, Employment, Deprivation, having visited a healthcare professional for mental health/alcohol/drug-related problems, BMI, nation, and stage at diagnosis

^c^An odds ratio greater than 1 represents a greater odds than the first group listed (reference category) of reporting psychological distress or poor mental well-being

The variable most strongly associated with poor mental well-being was reporting problems with self-care (OR 3.89; 95% CI 3.39 to 4.48), followed by reporting clinical levels of fatigue (OR 3.86; 3.38 to 4.42), problems with usual activities (OR 3.37; 95% CI 2.94 to 3.86), and mobility (OR 2.81; 95% CI 2.46 to 3.21). All cancer-related symptom bother and HRQL variables were significantly associated with psychological distress and poor mental well-being.

In the distress model, the strongest association was with reporting clinically significant fatigue (OR 9.92; 95% CI 7.63 to 12.89). Reporting problems with usual activities (OR 6.42; 95% CI 5.00 to 8.24), mobility (OR 4.51; 95% CI 3.61 to 5.63), and bother associated with bowel function (OR 3.46; 95% CI 2.83 to 4.23) were all associated with distress.

Results using multiple imputation were comparable to complete case analysis (Online Resources 3, 5).

## Discussion

This large study of just over 13,000 men diagnosed with prostate cancer who reported receiving ADT either alone or combined with other treatment, included men with all stages of disease at diagnosis. To our knowledge, this is one of the largest patient-reported outcome studies of men treated with ADT and therefore provides further insight into the relationship between cancer-related symptoms and psychological outcomes. As a population-based sample, not limited by stage of disease or specific ADT treatment, it therefore provides a general picture of psychological outcomes, using validated measures in men treated with ADT. The results demonstrate that while most men did not report poor psychological outcomes 18–42 months post diagnosis, a small percentage of men reported poor mental well-being (15.5%) or severe psychological distress (6.6%). After controlling for sociodemographic and clinical variables, all cancer-related symptoms and HRQL were significantly associated with both poor mental well-being and psychological distress. Reporting clinically significant levels of fatigue was most strongly associated with psychological distress and was also strongly associated with poor mental well-being compared to men without this level of fatigue.

The findings therefore suggest that experiencing high levels of fatigue is a possible indicator of poor mental health. However, at present, prostate cancer follow-up consultations are often focused on cancer-specific symptoms such as urinary and bowel function. Fatigue and/or distress can therefore be overlooked and not directly enquired about, despite their prevalence in cancer patients, particularly in men from high-risk groups such as men treated with ADT [36]. Men treated with ADT may be particularly vulnerable to poor psychological outcomes during and following treatment, yet may struggle to raise the issue of their mental health during clinical encounters. Men may also have difficulty in discussing the side effects of treatment, particularly due to the sensitive nature of the bodily changes that occur as part of ADT treatment (Matheson et al. submitted).

The men treated with ADT were compared to those treated without ADT (Online Resource 7). Cancer-related symptoms were reported by a greater proportion of the men treated with ADT, in particular, fatigue levels which are consistent with existing literature [6]. The levels of distress and poor well-being reported were also significantly higher in the group treated with ADT. These results support the focus of this study being on these particularly vulnerable men.

The development of the K6 included cross-validation with other measures of mental illness and assessment of its applicability as a screening tool [28, 29]. Although the K6 is not a diagnostic tool, the cut-off used in this study has been demonstrated as an indicator of possible severe mental illness [37]. The finding that a minority of men treated with ADT reported poor psychological outcomes is consistent with the literature, although the proportions reporting psychological distress were smaller than previous reports of anxiety [13]. This may be a consequence of the 18–42-month post-diagnosis timeframe used in the present study. The study findings demonstrate that a small proportion of men treated with ADT reported experiencing both clinically significant levels of fatigue as indicated by the EORTC-fatigue subscale, along with clinically significant psychological distress. This highlights the potentially unmet needs in these men.

The results from the present study support the routine use of tools to assess fatigue and/or psychological distress in clinical consultations or health needs assessments, which could help prompt earlier referral for intervention to help with the experience of cancer-related fatigue (e.g. supervised exercise, clinical psychology input, anti-depressants) [38–40]. These findings are particularly timely as over the past year, treatment for locally advanced and metastatic prostate cancer with ADT has become more intensive [41]. Chemotherapy is now used up front with ADT and the routine use of radiotherapy in men with limited metastatic disease may become a standard of care following recent data from the UK MRC STAMPEDE trial [42, 43].

The results would support incorporating specific advice on managing fatigue and risks of poor psychological outcomes in men treated with ADT into the education and supported self-management events that are currently being implemented as part of the recovery and rehabilitation programmes. Although many men treated with radiotherapy and ADT receive only short-term hormone deprivation therapy (circa 6 months), it can take 12–24 months before any related symptoms resolve and up to 10% may remain castrate with on-going problems [44, 45]. Often men with prostate cancer (and their health teams) are unaware of the risk of the continuing symptoms despite stopping ADT [46].

### Strengths and limitations

Reported here is a large-scale study of men diagnosed with prostate cancer and administered with ADT, not restricted by age or stage, representing one of the largest samples of men diagnosed with prostate cancer on ADT and providing important new data to better understand the experiences of these men. Validated measures of mental well-being and psychological distress were used to investigate these outcomes at 18–42 months post diagnosis. Additionally, the questionnaire response rate was 61%, which is typical for health-related surveys of this kind [47].

A key limitation of the present study is the cross-sectional nature of the data which means that causation cannot be inferred. The data also refer to a single time-point following diagnosis and treatment, which limits our understanding of the men’s experience over time. There was a lack of a baseline measure of either mental well-being or distress; although we did ask about previous mental health/alcohol-related contact with health services. It is, therefore, not possible to know precisely the progression of well-being/distress after treatment, or whether these were present before the start of treatment. It was also beyond the scope of this research to collect general population comparison data from across the UK and we only know the outcomes of the responding men. These factors limit the interpretation of the results to an extent, and caution should be taken when generalising the findings as longitudinal data are needed to confirm the findings. However, as a result of the 61% response rate and the large sample size, the present findings remain the largest of their kind and add to our understanding of these outcomes in men treated with ADT.

The time period of 18–42 months was selected as it represents the period when initial treatment is complete and side effects and quality of life have begun to stabilise. However, the survey did not collect data on the specific time since diagnosis of each respondent, and this is a limitation of the study. While the relationship between increased duration of ADT treatment and poorer quality of life is known [48], we did not know the duration of ADT treatment or whether the men were still in receipt of ADT at the time of the survey [13]. There is therefore likely variability in the respondents’ experiences of receiving ADT, both in duration and dose, which was not able to be controlled for in the multivariable analyses. In addition to this, the specific type of combination treatment was not included as an explanatory variable, due to the small size of a number of these subgroups. Due to this, we are not able to provide further insight as to whether certain treatment groupings had greater association with poor psychological outcomes.

There are a number of avenues for future research based on these findings. Further research is needed to study ways of dealing with the adverse effects of ADT. As mentioned previously, there is some evidence for physical activity interventions in reducing fatigue [38, 39, 49] although the impact of physical activity on outcomes in men with prostate cancer is incompletely understood, particularly in relation to mental health. Further high-level research is needed, in part this is currently being tested in a formal randomised trial setting in the UK (the ‘STAMINA’ study [50]), but results will not be available for some time. Mechanistically, physical activity might be expected to ameliorate the cardiovascular adverse effects of ADT, just as it does in other settings such as diabetes or established cardiovascular disease.

## Conclusions

While most men treated with ADT living 18–42 months following a diagnosis of PCa do not report poor mental well-being or severe psychological distress, a small proportion report poor psychological outcomes. Reporting clinically significant levels of fatigue was found to be a clear indicator of psychological distress. These results support the need to enquire about fatigue and distress more directly as part of the clinical interaction [51] and for healthcare systems to have clear protocols in place which specifically and routinely target both psychological distress and fatigue.

## Electronic supplementary material

Below is the link to the electronic supplementary material.

Supplementary material 1 (PDF 1091 kb)

Supplementary material 2 (DOCX 41 kb)
