# Supplementary material for: Cancer-related symptoms, mental well-being, and psychological distress in men diagnosed with prostate cancer treated with androgen deprivation therapy
Source: Qual Life Res. 2019 May 21;28(10):2741–51. doi: 10.1007/s11136-019-02212-x (PMC6761086; doi:10.1007/s11136-019-02212-x)
Supplement: Supplementary file 1 — Supplementary material 1 (PDF 1091 kb) [file 11136_2019_2212_MOESM1_ESM.pdf]

Article title: Cancer-related symptoms, mental well-being and psychological distress in men diagnosed with prostate cancer treated with Androgen Deprivation Therapy.

Journal: Quality of Life Research.

Authors: Sarah Wilding (corresponding author) S.E.Wilding@leeds.ac.uk; University of Leeds, Amy Downing, Penny Wright, Peter Selby, Eila Watson, Richard Wagland, David W Donnelly, Luke Hounsome, Hugh Butcher, Malcolm Mason, Ann Henry, Anna Gavin, Adam W Glaser.

Online resource 1. Full questionnaire

Reference Number

Life After Prostate Cancer Diagnosis: Patient Reported Outcomes Survey

More men are now living longer after a diagnosis of prostate cancer. We want to find out what life is really like for this group of men. Your answers will benefit other men with prostate cancer in the future by providing information to help clinical teams, service providers and policy makers make decisions about how to improve the quality of services for prostate cancer patients. We would be grateful if you would complete this survey, which asks for information about your health and quality of life.

If you have **not** had a diagnosis of prostate cancer this questionnaire is not relevant to you. Please tick the "no" box below and please accept our apologies for contacting you. Please return the blank questionnaire in the envelope provided and we will correct our records. If you have any questions about this survey please contact the FREEPHONE helpline number: **0808 801 0678**.

Have you ever been told by a doctor that you have prostate cancer?

Yes ☐ No ☐

If you have ticked yes to the first question please complete the rest of the survey.

The survey
This survey is made up of eight sections and will take approximately 30 minutes to complete.

Who should complete the questionnaire?
The questions should be answered by the person named in the letter that came with this questionnaire. If that person needs help to answer the questions then the answers should be given from their point of view – not from the point of view of the person who is helping.

Completing the questionnaire
For each question please tick clearly inside the box of the response that best represents your views, using a black or blue pen. Do not worry if you make a mistake. Just cross out the mistake and put a tick in the correct box. Do not write your name or address anywhere on the questionnaire. The more questions in this survey that you complete, the more we can understand what life is like for those living with and beyond prostate cancer. However, if you feel unable or uncomfortable about answering any of the questions, leave it blank and move on to the next one.

The information you give us will be kept **securely** and treated in **confidence**. We will not publish any personal information that could allow anyone to identify you. We are very grateful for your time and effort in completing this survey.

If you have any queries about the questionnaire, please call the FREEPHONE helpline number: **0808 801 0678**

You can find more information about the study at:
http://www.lifeafterprostatecancerdiagnosis.com/

Section One: Your overall health

Under each heading, please tick ONE box that best describes your health TODAY

1. MOBILITY

I have no problems in walking about ❑
I have slight problems in walking about ❑
I have moderate problems in walking about ❑
I have severe problems in walking about ❑
I am unable to walk about ❑

2. SELF-CARE

I have no problems washing or dressing myself ❑
I have slight problems washing or dressing myself ❑
I have moderate problems washing or dressing myself ❑
I have severe problems washing or dressing myself ❑
I am unable to wash or dress myself ❑

3. USUAL ACTIVITIES (e.g. work, study, housework, family or leisure activities)

I have no problems doing my usual activities ❑
I have slight problems doing my usual activities ❑
I have moderate problems doing my usual activities ❑
I have severe problems doing my usual activities ❑
I am unable to do my usual activities ❑

4. PAIN / DISCOMFORT

I have no pain or discomfort ❑
I have slight pain or discomfort ❑
I have moderate pain or discomfort ❑
I have severe pain or discomfort ❑
I have extreme pain or discomfort ❑

5. ANXIETY / DEPRESSION

I am not anxious or depressed ❑
I am slightly anxious or depressed ❑
I am moderately anxious or depressed ❑
I am severely anxious or depressed ❑
I am extremely anxious or depressed ❑

Are there any additional HEALTH issues that are of concern to you?

© 1998 EuroQol GroupEQ-5D™ is a trade mark of the EuroQol Group

6. We would like to know how good or bad your health is TODAY.

- This scale is numbered from 0 to 100.
- 100 means the best health you can imagine.
  0 means the worst health you can imagine.
- Mark an X on the scale to indicate how your health is TODAY.
- Now, please write the number you mark on the scale in the box below.

The best health

you can imagine

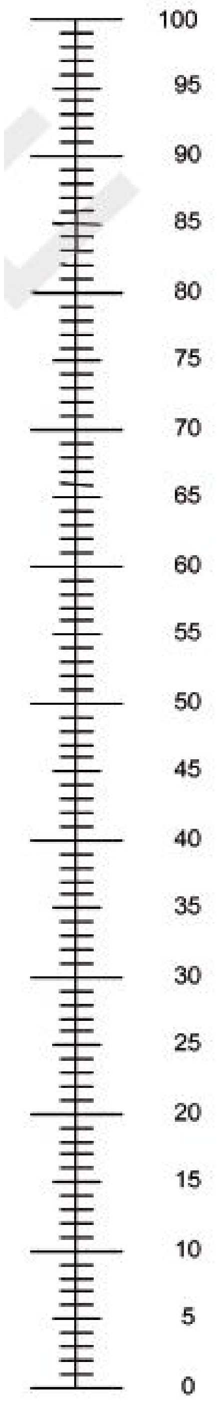

YOUR HEALTH TODAY =

Section Two: Your diagnosis and treatment

7. How were you diagnosed? *Please tick **all that apply***

☐ I attended my GP with urinary symptoms (e.g. urinating frequently, blood in urine)

☐ I attended my GP with other symptoms (e.g. back pain, joint pain)

☐ I had no symptoms and my GP offered to test my PSA (blood test) as part of a general health check

☐ I had no symptoms and I asked my GP to measure my PSA (blood test)

☐ I had symptoms and I asked my GP to measure my PSA (blood test)

☐ I had a PSA test as part of a private health check

☐ Other — Please tell us about this:

8. Please tell us which treatments you have had following your diagnosis of prostate cancer
*Please tick **all** the options that apply.*

A. Have you had surgery (prostatectomy)? No ☐ Yes ☐

If **no**, go to **B**

If **yes**, what type of surgery? Please tick one box

Open prostatectomy ☐
Operation performed through a cut in the abdomen above the pubic bone area (retropubic prostatectomy) or a cut in the area between the testicles and back passage (perineal prostatectomy).

Laparoscopic (keyhole) prostatectomy ☐
Operation performed through small incisions in the abdominal wall.

Robotic prostatectomy ☐
Operation performed with the assistance of a surgical robot (Da Vinci prostatectomy).

I don't know what kind of operation I had ☐

B. Have you had radiotherapy? No ☐ Yes ☐

If **no**, go to **C**

If **yes**, what type of radiotherapy? Please tick all that apply

External beam radiotherapy (with or without hormone treatment) ☐
Radiotherapy uses high-energy X-ray beams to treat the whole prostate. This form of treatments includes both 3-dimensional conformal radiotherapy (3D-CRT) and intensity modulated radiotherapy (IMRT).

Permanent seed (low-dose) brachytherapy ☐
This involves implanting radioactive seeds into the prostate gland.

Temporary (high-dose) brachytherapy (with or without external beam radiotherapy or hormone treatment) ☐
This involves inserting a source of high-dose radiation into the prostate gland for a few minutes.

I don't know what type of radiotherapy or brachytherapy I had ☐

C. Have you had any of the following treatments?

Please tick all that apply.

High intensity focused ultrasound (HIFU) ☐
This treatment uses ultrasound waves to heat and destroy cancer cells in the prostate.

Cryotherapy ☐
This treatment uses freezing and thawing to kill the cancer cells in the prostate.

Chemotherapy (not including hormones) ☐

Hormone treatment (either continuous or on/off treatment) ☐

Abiraterone and/or Enzalutamide ☐

D. Are doctors and nurses currently monitoring <u>your</u> prostate cancer?

No ☐ Yes ☐

If **no**, go to question **9**

If **yes**, what type of monitoring? Please tick one box.

Active Surveillance ☐
Surveillance is monitoring of low risk, slow growing ***localised prostate cancer*** *with the aim of avoiding or delaying* ***curative treatment*** *(e.g. surgery, radiotherapy).This involves having regular tests.*

Watchful waiting ☐
Watchful waiting is a way of monitoring prostate cancer that isn't causing any symptoms or problems. The aim is to keep an eye on the cancer over the long term and only having treatment if the cancer deteriorates or the patient gets symptoms. This involves fewer tests than in active surveillance.

Clinical follow-up during or after one of the treatments mentioned above in 8A, B or C ☐

I am unsure about the type of monitoring I am currently having ☐

9a. Do you think your views were taken into account when the team of doctors and nurses caring for you were discussing which treatment you should have? Please tick one of the following boxes:

- ☐ Yes, definitely
- ☐ Yes, to some extent
- ☐ No, my views were not taken into account
- ☐ I didn't know my treatment was being discussed by a team of doctors / nurses
- ☐ Not sure / can't remember

9b. Please answer the following questions whether or not you were actively involved in the decisions made about your treatment.

Please think about the decisions you made about your treatment for prostate cancer after talking to your doctor, surgeon, nurse, health care professional etc.

Please show how you feel about these statements by ticking ***one*** *box on each row.*

	Strongly agree	Agree	Neither agree nor disagree	Disagree	Strongly disagree
a. It was the right decision	☐	☐	☐	☐	☐
b. I regret the choice that was made	☐	☐	☐	☐	☐
c. I would go for the same choice if I had to do it over again	☐	☐	☐	☐	☐
d. The choice did me a lot of harm	☐	☐	☐	☐	☐
e. The decision was a wise one	☐	☐	☐	☐	☐

Please add anything else you would like to tell us about your diagnosis, treatment, and the decision making process.

Section Three: How things are for you now

We understand that some of the following questions are very sensitive, but we would really appreciate you answering them if possible. As with the rest of the questionnaire, your answers will be kept confidential and no one will be able to identify you.

*Please tick **one** box for each question.*

10.	Over the past 4 weeks, how often have you leaked urine?
More than once a day	☐
About once a day	☐
More than once a week	☐
About once a week	☐
Rarely or never	☐

11.	Which of the following best describes your urinary control during the last 4 weeks?
No urinary control whatsoever	☐
Frequent dribbling	☐
Occasional dribbling	☐
Total control	☐

12.	How many pads per day did you usually use to control leakage during the last 4 weeks?
None	☐
1 pad per day	☐
2 pads per day	☐
3 or more pads per day	☐

13.	How big a problem, if any, has each of the following been for you during the last 4 weeks? *Please tick one box on each line.*				
	No problem	Very small problem	Small problem	Moderate problem	Big problem
a. Dripping or leaking urine	☐	☐	☐	☐	☐
b. Pain or burning on urination	☐	☐	☐	☐	☐
c. Bleeding with urination	☐	☐	☐	☐	☐
d. Weak urine stream or incomplete emptying	☐	☐	☐	☐	☐
e. Need to urinate frequently during the day	☐	☐	☐	☐	☐

14.	Overall, how big a problem has your urinary function been for you during the last 4 weeks? *Please tick one box.*
No problem	☐
Very small problem	☐
Small problem	☐
Moderate problem	☐
Big problem	☐

15.	How big a problem, if any, has each of the following been for you during the last 4 weeks? *Please tick one box on each line.*				
	No problem	Very small problem	Small problem	Moderate problem	Big problem
a. Urgency to have a bowel movement	☐	☐	☐	☐	☐
b. Increased frequency of bowel movements	☐	☐	☐	☐	☐
c. Losing control of your stools	☐	☐	☐	☐	☐
d. Bloody stools	☐	☐	☐	☐	☐
e. Abdominal/ Pelvic/Rectal pain	☐	☐	☐	☐	☐

16.	Overall, how big a problem have your bowel habits been for you during the last 4 weeks? *Please tick one box.*
No problem	
Very small problem	
Small problem	
Moderate problem	
Big problem	

17.	How would you rate each of the following during the last 4 weeks? *Please tick one box on each line.*				
	Very poor to none	Poor	Fair	Good	Very good
a. Your ability to have an erection					
b. Your ability to reach orgasm (climax)					

18.	How would you describe the usual QUALITY of your erections during the last 4 weeks? *Please tick one box.*
None at all	
Not firm enough for any sexual activity	
Firm enough for masturbation and foreplay only	
Firm enough for intercourse	

*Please tick **one** box for each question.*

19.	How would you describe the FREQUENCY of your erections during the last 4 weeks?
I NEVER had an erection when I wanted one	☐
I had an erection LESS THAN HALF the time I wanted one	☐
I had an erection ABOUT HALF the time I wanted one	☐
I had an erection MORE THAN HALF the time I wanted one	☐
I had an erection WHENEVER I wanted one	☐

20.	Overall, how would you rate your ability to function sexually during the last 4 weeks?
Very poor	☐
Poor	☐
Fair	☐
Good	☐
Very good	☐

21.	Overall, how big a problem has your sexual function or lack of sexual function been for you during the last 4 weeks?
No problem	☐
Very small problem	☐
Small problem	☐
Moderate problem	☐
Big problem	☐

22.	How big a problem during the last 4 weeks, if any, has each of the following been for you? *Please tick one box on each line.*				
	No problem	Very small problem	Small problem	Moderate problem	Big problem
Hot flushes	☐	☐	☐	☐	☐
Breast tenderness/enlargement	☐	☐	☐	☐	☐
Feeling depressed	☐	☐	☐	☐	☐
Lack of energy	☐	☐	☐	☐	☐
Change in body weight	☐	☐	☐	☐	☐

**During the last 4 weeks** *Please tick **one** box on each line.*				
	Not at all	A little	Quite a bit	Very much
**23.** To what extent were you interested in sex?	☐	☐	☐	☐
**24**. To what extent were you sexually active (with or without intercourse)?	☐	☐	☐	☐

25. Have you used any medications to aid or improve erections since your prostate cancer diagnosis? (e.g. tablets, penis injections, gels) *Please tick one box.*						
☐	☐	☐	☐	☐	☐	☐
**I was not offered this**	I was offered this but did not want it	I was offered this but have not tried it	I was offered this and tried it, but it	I was offered this and it helped, but I am	I was offered this, it helps and I use it	I was offered this, it helps

			was not helpful	not using it now	sometimes	and I use it often

26. Have you used any devices to aid or improve erections since your prostate cancer diagnosis? (e.g. vacuum pump, penile prosthesis) ***Please tick one box.***

☐	☐	☐	☐	☐	☐	☐
**I was not offered this**	I was offered this but did not want it	I was offered this but have not tried it	I was offered this and tried it, but it was not helpful	I was offered this and it helped, but I am not using it now	I was offered this, it helps and I use it sometimes	I was offered this, it helps and I use it often

27. Have you used any specialist services to help with your sex life following your diagnosis of prostate cancer? (e.g. counselling, psychosexual clinics, psychology)
Please tick one box.

☐	☐	☐	☐	☐	☐
**I was not offered this**	I was offered this but did not want it	I was offered this but have not tried it	I was offered this and tried it, but it was not helpful	I was offered this and it helped, but I am not using it now	I was offered this, it helps and I am still using the service

During the past week: *Please tick* ***one*** *box on each line.*

	Not at all	A little	Quite a bit	Very much
**28.** Did you need to rest?	☐	☐	☐	☐
**29.** Have you felt weak?	☐	☐	☐	☐
**30.** Were you tired?	☐	☐	☐	☐

Please add anything else you would like to tell us about your symptoms or the side effects of your treatment.

Section Four: Your everyday life

On each line please tick the box that best describes your answer.

Please tick the **'no difficulty box'** if a question **does not apply to you**.

During the past month:	No difficulty	A little difficulty	Quite a bit of difficulty	Very much difficulty
**31.** Have you had any difficulty maintaining your independence?	☐	☐	☐	☐
**32.** Have you had any difficulty in carrying out your domestic chores? (e.g. cleaning, gardening, cooking, shopping)	☐	☐	☐	☐
**33.** Have you had any difficulty with managing your own personal care? (e.g. bathing, dressing, washing)	☐	☐	☐	☐
**34.** Have you had any difficulty with looking after those who depend on you? (e.g. children, dependent adults, pets)	☐	☐	☐	☐
**35.** Have any of those close to you (e.g. partner, children, parents) had any difficulty with the support available to them?	☐	☐	☐	☐
**36.** Have you had any difficulties with benefits? (e.g. Statutory Sick Pay, Personal Independence Payments, Attendance Allowance, Universal Credit)	☐	☐	☐	☐
**37.** Have you had any financial difficulties?	☐	☐	☐	☐
**38.** Have you had any difficulties with financial services? (e.g. loans, mortgages, pensions, insurance)	☐	☐	☐	☐
**39.** Have you had any difficulty concerning your work? (or education if you are a student)	☐	☐	☐	☐
**40.** Have you had any difficulty with planning for your own or your family's future? (e.g. care of dependents, legal issues, business affairs)	☐	☐	☐	☐

	No difficult y	A little difficult y	Quite a bit of difficult y	Very much difficult y
**41.** Have you had any difficulty with communicating with those closest to you? (e.g. partner, children ,parents)	☐	☐	☐	☐
During the past month:				
**42.** Have you had any difficulty with communicating with others? (e.g. friends, neighbours, colleagues, dates)	☐	☐	☐	☐
**43.** Have you had any difficulty concerning plans to have a family?	☐	☐	☐	☐
**44.** Have you had any difficulty concerning your appearance or body image?	☐	☐	☐	☐
**45.** Have you felt isolated?	☐	☐	☐	☐
**46.** Have you had any difficulty with getting around? (e.g. transport, car parking, your mobility)	☐	☐	☐	☐
**47.** Have you had any difficulty in carrying out your recreational activities (e.g. hobbies, pastimes, social pursuits)	☐	☐	☐	☐
**48.** Have you had any difficulty with your plans to travel or take a holiday?	☐	☐	☐	☐

49. In the **past week**, on how many days have you done a total of 30 minutes or more of physical activity, which was enough to raise your heart rate?
(This may include sport, exercise and brisk walking or cycling for recreation or to get to and from places, ***but should not include housework or physical activity that is part of your job.)***
Please tick ***one*** *box.*

☐	☐	☐	☐	☐	☐	☐	☐
**None**	**I day**	**2 days**	**3 days**	**4 days**	**5 days**	**6 days**	**7 days**

Please add anything else you would like to tell us about how your prostate cancer has had an impact on your everyday life.

Section Five: Your emotional wellbeing

Below are some statements about feelings and thoughts. Please tick the box on each line that best describes your experience of each over **the last 2 weeks.**

STATEMENTS	None of the time	Rarely	Some of the time	Often	All of the time
**50.** I've been feeling optimistic about the future	☐	☐	☐	☐	☐
**51.** I've been feeling useful	☐	☐	☐	☐	☐
**52.** I've been feeling relaxed	☐	☐	☐	☐	☐
**53.** I've been dealing with problems well	☐	☐	☐	☐	☐
**54.** I've been thinking clearly	☐	☐	☐	☐	☐
**55.** I've been feeling close to other people	☐	☐	☐	☐	☐
**56.** I've been able to make up my own mind about things	☐	☐	☐	☐	☐

The following questions ask about how you have been feeling during the **past 30 days**.
For each question, please tick the box on each line that best describes how often you had this feeling.
During the past 30 days, about how often did you feel…

	All of the time	Most of the time	Some of the time	A little of the time	None of the time
**57. ...nervous?**	☐	☐	☐	☐	☐
**58. ...hopeless?**	☐	☐	☐	☐	☐
**59. ...restless or fidgety?**	☐	☐	☐	☐	☐
**60. ...so depressed that nothing could cheer you up?**	☐	☐	☐	☐	☐
**61. ...that everything was an effort?**	☐	☐	☐	☐	☐
**62. ...worthless?**	☐	☐	☐	☐	☐

Please add anything else you would like to tell us about how your prostate cancer has had an impact on your emotional well-being.

Section Six: Looking to the future

Even if you are now free from prostate cancer please complete this section.

Please read the statements carefully and tick your responses to them. *Please tick* ***one*** *box on each line.* **If a question does not apply to you please leave it blank.**

	Strongly agree	Agree	Disagree	Strongly disagree
**62.** I am capable of coping with my prostate cancer	☐	☐	☐	☐
**63.** I have all the information I need to manage my prostate cancer	☐	☐	☐	☐
**64.** I am capable of helping health professionals reach decisions related to my prostate cancer	☐	☐	☐	☐
**65.** My family are very supportive	☐	☐	☐	☐
**66.** I need the support of my family and friends	☐	☐	☐	☐
**67.** My family and friends still rely on me	☐	☐	☐	☐
**68.** I can adapt to the changes in my lifestyle	☐	☐	☐	☐
**69.** Health professionals are happy to include me in decisions related to my prostate cancer	☐	☐	☐	☐
**70.** I want my family and friends to continue to rely on me	☐	☐	☐	☐
**71.** My friends are always supportive	☐	☐	☐	☐
**72.** I still feel useful in my daily life	☐	☐	☐	☐
**73.** My spiritual beliefs help me cope with my prostate cancer	☐	☐	☐	☐
**74.** I accept that I have to change my lifestyle	☐	☐	☐	☐
**75.** Complementary therapies help me cope with my prostate cancer	☐	☐	☐	☐
**76.** I have a lot of confidence in my local GP	☐	☐	☐	☐
	No impact	A little impact	Quite a bit of impact	Very much impact
**77.** How much of an impact has prostate cancer had on your life?				

Section Seven: Questions about you

78. How old are you (in years)?

☐

79. What is your **legal** marital status? *Please tick* ***one*** *box.*

- ☐ Married
- ☐ In civil partnership
- ☐ Separated
- ☐ Divorced/dissolved civil partnership
- ☐ Widowed/surviving partner from civil partnership
- ☐ Single (never married/never in civil partnership)
- ☐ Other

80. What was your employment status before your diagnosis of prostate cancer? *Please tick* ***one*** *box.*

- ☐ Full time employment
- ☐ Part time employment
- ☐ Self employed
- ☐ Looking after family/home
- ☐ Retired
- ☐ Unemployed, seeking work
- ☐ Unemployed, unable to work for health reasons
- ☐ Other

What have been the most important issues that you have faced since your prostate cancer diagnosis?

- ☐
- ☐
- ☐
- ☐
- ☐ [illegible]
- ☐ Unemployed, seeking work
- ☐ Unemployed, unable to work for health reasons
- ☐ Other

82. To which of these ethnic groups would you say you belong? *Please tick **one** box.*

White

- ☐ English/Welsh/Scottish/Northern Irish/British
- ☐ Irish
- ☐ Gypsy or Irish Traveller
- ☐ Any other White background

Mixed/Multiple ethnic groups

- ☐ White and Black Caribbean
- ☐ White and Black African
- ☐ White and Asian
- ☐ Any other Mixed/multiple ethnic background

Asian / British Asian

- ☐ Indian
- ☐ Pakistani
- ☐ Bangladeshi
- ☐ Chinese
- ☐ Any other Asian background

Black/African/Caribbean/Black British

- ☐ Black African
- ☐ Black Caribbean
- ☐ Any other Black / African / Caribbean background

Other ethnic group

- ☐ Arab
- ☐ Any other ethnic group

83. Do you consider yourself… *Please tick **one** box.*

- ☐ Heterosexual / straight
- ☐ Homosexual / gay
- ☐ Bisexual
- ☐ Don't know
- ☐ Prefer not to answer

84. Which, if any, of the following conditions do you have? *Please tick **all** the boxes that apply.*

- ☐ A heart condition
- ☐ Angina
- ☐ High blood pressure
- ☐ Asthma or other chronic chest problem
- ☐ Liver disease
- ☐ Problems with your stomach, bowels or gallbladder
- ☐ Problems with your pancreas

- ☐ Kidney disease
- ☐ Diabetes
- ☐ Stroke
- ☐ Alzheimer's disease or dementia
- ☐ Epilepsy
- ☐ Other long standing neurological problem
- ☐ A diagnosis of arthritis

85. How tall are you? ……feet…….inches OR ……centimetres ☐ Don't know

86. How much do you weigh? …….stone……pounds OR ….kilograms… grams ☐ Don't know

87. Have you <u>ever in your lifetime</u> seen a health care professional (such as a GP, psychiatrist, psychologist, social worker, counsellor, psychotherapist, mental health nurse, or any other such professional) for problems with your emotions or nerves or your use of alcohol or drugs?

☐ Yes ☐ No

88. Do you look after, or give any help or support (not part of your paid employment) to family members, friends, neighbours or others because of either:

- Long term physical or mental health disability, or
- Problems relating to old age

☐ Yes ☐ No

Is there anything else you would like to tell us about what life has been like for you following your prostate cancer diagnosis?

Please would you tell us who filled in this survey? *Please tick **one** box.*

☐ The person to whom this survey was sent

☐ A representative of the person to whom this survey was sent
(e.g. partner, family member, friend)

Section Eight: Future contact

Follow-up survey

Thank you for completing this survey. We will be **contacting you again in a years time** for the follow-up survey. If you decide at that time you would rather not complete the survey again there is no obligation to do so.

Follow-up interview

As part of this work we are going to be interviewing a small number of men and some partners /spouses to gain a better understanding of their experience. This will only involve men and their partners / spouses who indicate they are interested in being interviewed. We would normally only be able to interview either the participant or their partner/spouse. We will not be interviewing couples together.

If you or your partner/spouse is interested in being involved in the interview, please tick the relevant boxes below.

☐ I am interested in being interviewed for this work

☐ My partner / spouse is interested in being interviewed for this work

We are sorry we will not be able to contact all those who would like to participate in the interview, but we will make sure that we get the views of a varied group. If you are one of the group we would like to interview we will write to you explaining how to get in touch with us to find out more about the interview. If you are still interested, we would arrange a time for the interview. If you think your partner/spouse would like to be interviewed, we would follow a similar process by making the initial contact with you by letter.

You have completed the survey.
Thank you for your time.

If you would like to know more about this study then please visit our website: http://www.lifeafterprostatecancerdiagnosis.com/

We *very much appreciate the time and thought you have put into completing this survey. If reflecting on your situation has caused anxiety or uncertainty in any way, please do not hesitate to contact your specialist cancer nurse or call one of the specialist nurses on Prostate Cancer UK's Confidential Help Line.*

Prostate Cancer UK's Confidential Help Line telephone: ***0800 074 8383***

(Free from UK landlines)

(Monday – Friday 9-6pm, Wednesday 10-8pm).

They are there to listen to your concerns, and offer support and helpful information
