# Supplementary material for: Cancer-related symptoms, mental well-being, and psychological distress in men diagnosed with prostate cancer treated with androgen deprivation therapy
Source: Qual Life Res. 2019 May 21;28(10):2741–51. doi: 10.1007/s11136-019-02212-x (PMC6761086; doi:10.1007/s11136-019-02212-x)
Supplement: Supplementary file 2 — Supplementary material 2 (DOCX 41 kb) [file 11136_2019_2212_MOESM2_ESM.docx]

Article title: Cancer-related symptoms, mental well-being and psychological distress in men diagnosed with prostate cancer treated with Androgen Deprivation Therapy.

Journal: Quality of Life Research.

Authors: Sarah Wilding (corresponding author) [S.E.Wilding@leeds.ac.uk](mailto:S.E.Wilding@leeds.ac.uk); University of Leeds, Amy Downing, Penny Wright, Peter Selby, Eila Watson, Richard Wagland, David W Donnelly, Luke Hounsome, Hugh Butcher, Malcolm Mason, Ann Henry, Anna Gavin, Adam W Glaser.

**Online Supplementary Resources**

**Online resource 1: Full questionnaire (see separate file)**

**Online resource 2. Treatment information**

**Online resource 3:** **Table presenting complete case analysis univariable and multivariable associations between socio-demographic and clinical factors and distress and well-being**

**Online resource 4. Table presenting characteristics of respondents reporting severe psychological distress and poor mental well-being.**

**Online resource 5. Table presenting complete case analysis. Multivariable associations between cancer-related symptoms, HRQL and psychological distress and mental well-being, when controlling for variables in the core models.**

**Online resource 6. Table presenting age, stage, cancer related symptoms, HRQL and psychological outcomes in men treated with ADT alone vs. treated with ADT and EBRT.**

**Online resource 7. Table presenting a comparison of cancer-related symptoms in men with and without ADT.**

**Online resource 2.**

A much higher than expected proportion of the overall sample (3.4%) reporting having High Intensity Focused Ultrasound (HIFU), indicating some confusion with this terminology. This group was excluded from analyses, as were men reporting having radiotherapy who did not know which type (4.6%) and those who reported unassigned combinations of treatment types (5.0%).

It was not clear that these men understood or were able to reliably report the treatment they had received, these men were also therefore excluded from the analyses focusing on men that reported receiving ADT.

|  |  | N | % |
| --- | --- | --- | --- |
| **Included in treatment-specific analyses** | **ADT treatments** |  |  |
|  | Androgen Deprivation Therapy (ADT) | 3,116 | 8.7 |
|  | EBRT + ADT | 7,488 | 20.9 |
|  | Surgery + EBRT & ADT | 901 | 2.5 |
|  | Surgery + ADT | 581 | 1.6 |
|  | Systemic therapy + ADT (+/EBRT) | 1,011 | 8.1 |
|  | **Treatments not including ADT** |  |  |
|  | Surgery | 7,054 | 19.7 |
|  | Active surveillance | 2,928 | 8.2 |
|  | External beam radiotherapy (EBRT) | 2,536 | 7.1 |
|  | Watchful waiting | 2,292 | 6.4 |
|  | Brachytherapy | 1,208 | 3.4 |
|  | Surgery + EBRT | 1448 | 4.0 |
|  | Systemic therapy + EBRT | 132 | 0.4 |
|  |  |  |  |
| **Excluded from treatment-specific analyses** | **Other:** |  |  |
|  | RT unknown type (alone or in combination) | 1,664 | 4.6 |
|  | HIFU (alone or in combination) | 1,231 | 3.4 |
|  | Follow-up only (unknown type) | 746 | 2.1 |
|  | No options ticked | 294 | 0.8 |
|  | Unassigned combination | 1,774 | 5.0 |
|  |  | **35,823** | **100** |

Systemic therapy: Chemotherapy/Abiraterone/Enzalutamide; RT: Radiotherapy; HIFU: High intensity focused ultrasound

**Online resource 3.** Complete case analysis univariable and multivariable associations between socio-demographic and clinical factors and distress and well-being.

|  |  | **Poor mental well-being (N = 9,306)** | | **Severe psychological distress (N = 9,787)** | |
| --- | --- | --- | --- | --- | --- |
|  |  | **OR (95% CI)** | | **OR (95% CI)** | |
| Characteristic | | **Univariable** | **Multivariable** | **Univariable** | **Multivariable** |
| Age | <55 years | 1.00 | 1.00 | 1.00 | 1.00 |
|  | 55-64 years | 1.28 (0.73-2.23) | 0.94 (0.49-1.80) | 0.77 (0.43-1.40) | 0.40 (0.19-0.86) |
|  | 65-74 years | 0.68 (0.40-1.18) | 0.74 (0.38-1.42) | 0.35 (0.19-0.62) | 0.30 (0.14-0.64) |
|  | 75-84 years | 0.83 (0.48-1.43) | 0.91 (0.47-1.76) | 0.28 (0.16-0.51) | 0.23 (0.11-0.52) |
|  | 85+ years | 1.23 (0.70-2.18) | 1.27 (0.63-2.58) | 0.46 (0.24-0.86) | 0.36 (0.15-0.85) |
| **Number of LTCs** | 0 | 1.00 | 1.00 | 1.00 | 1.00 |
|  | 1 | 1.13 (0.97-1.30) | 1.08 (0.90-1.29) | 1.22 (0.96-1.56) | 1.25 (0.92-1.68) |
|  | 2 | 1.64 (1.41-1.91) | 1.54 (1.28-1.86) | 2.13 (1.67-2.71) | 2.05 (1.52-2.79) |
|  | 3 | 2.29 (1.93-2.72) | 2.07 (1.67-2.56) | 3.27 (2.53-4.23) | 3.26 (2.35-4.53) |
|  | 4+ | 3.03 (2.54-3.63) | 2.79 (2.23-3.50) | 6.17 (4.81-7.92) | 5.47 (3.95-7.57) |
| **Employment** | Employed | 1.00 | 1.00 | 1.00 | 1.00 |
|  | Unemployed | 5.44 (4.10-7.20) | 3.25 (2.29-4.62) | 9.34 (6.66-13.10) | 6.09 (3.91-9.49) |
|  | Retired | 1.15 (0.99-1.34) | 1.01 (0.83-1.24) | 1.27 (1.00-1.60) | 1.55 (1.11-2.17) |
|  | Other | 3.07 (1.67-5.66) | 2.04 (0.92-4.53) | 2.20 (0.85-5.68) | 1.25 (0.29-5.47) |
| **Ethnicity** | White | 1.00 | 1.00 | 1.00 | 1.00 |
|  | Non-white | 1.17 (0.83-1.63) | 0.73 (0.46-1.18) | 1.62 (1.06-2.46) | 0.90 (0.48-1.69) |
| **Marital status** | Married/Civil Partner | 1.00 | 1.00 | 1.00 | 1.00 |
|  | Separated/Divorced | 1.69 (1.42-2.01) | 1.38 (1.11-1.72) | 2.04 (1.63-2.56) | 1.35 (1.00-1.83) |
|  | Widowed | 1.55 (1.32-1.82) | 1.24 (1.00-1.53) | 1.21 (0.94-1.55) | 1.22 (0.88-1.68) |
|  | Single | 1.50 (1.18-1.91) | 1.10 (0.81-1.51) | 1.19 (0.82-1.72) | 0.80 (0.49-1.31) |
|  | Other | 1.44 (0.94-2.20) | 1.13 (0.67-1.92) | 1.64 (0.94-2.86) | 1.12 (0.52-2.44) |
| **Deprivation quintile** | least deprived | 1.00 | 1.00 | 1.00 | 1.00 |
|  | 2 | 1.18 (1.02-1.36) | 1.21 (1.02-1.44) | 1.25 (0.99-1.56) | 1.35 (1.00-1.83) |
|  | 3 | 1.26 (1.08-1.46) | 1.27 (1.06-1.51) | 1.57 (1.25-1.97) | 1.22 (0.88-1.68) |
|  | 4 | 1.67 (1.42-1.95) | 1.42 (1.17-1.72) | 2.42 (1.92-3.04) | 0.80 (0.49-1.31) |
|  | most deprived | 2.40 (2.02-2.84) | 1.90 (1.53-2.35) | 3.42 (2.68-4.35) | 1.12 (0.52-2.44) |
| **Carer status** | No | 1.00 | 1.00 | 1.00 | 1.00 |
|  | Yes | 1.05 (0.94-1.18) | 1.06 (0.92-1.21) | 1.11 (0.95-1.31) | 1.18 (0.96-1.43) |
| **Mental Health help seeking** | No | 1.00 | 1.00 | 1.00 | 1.00 |
|  | Yes | 2.39 (2.13-2.68) | 2.23 (1.94-2.57) | 3.77 (3.25-4.39) | 3.03 (2.51-3.66) |
| **Stage** | I/II | 1.00 | 1.00 | 1.00 | 1.00 |
|  | III | 1.05 (0.93-1.19) | 1.14 (0.99-1.31) | 1.12 (0.94-1.34) | 1.27 (1.03-1.56) |
|  | IV | 1.21 (1.06-1.38) | 1.26 (1.09-1.46) | 1.25 (1.04-1.51) | 1.36 (1.09-1.70) |
| **BMI** | <25 Under/healthy | 1.00 | 1.00 | 1.00 | 1.00 |
|  | 25-30 Overweight | 0.83 (0.75-0.95) | 0.86 (0.75-1.00) | 0.91 (0.75-1.10) | 0.84 (0.67-1.05) |
|  | 30+ Obese | 1.33 (1.09-1.52)- | 1.18 (1.00-1.39) | 1.69 (1.39-2.05) | 1.15 (0.90-1.46) |
| **Nation** | England | 1.00 | 1.00 | 1.00 | 1.00 |
|  | Wales | 1.20 (1.00-1.44) | 1.15 (0.92-1.43) | 1.39 (1.08-1.77) | 1.30 (0.95-1.78) |
|  | Scotland | 1.27 (1.03-1.57) | 1.24 (0.95-1.61) | 1.41 (1.06-1.88) | 1.43 (1.00-2.06) |
|  | NI | 1.08 (0.80-1.44) | 1.01 (0.72-1.41) | 1.41 (0.96-2.06) | 1.15 (0.71-1.86) |

OR odds ratios, CI 95% confidence intervals

**Online resource 4.** Characteristics of respondents reporting severe psychological distress and poor mental well-being. Numbers and percentages and p values^a^

|  | **Mental well-being** | | | | | | **Psychological distress** | | | | | |  |
| --- | --- | --- | --- | --- | --- | --- | --- | --- | --- | --- | --- | --- | --- |
| **Characteristics** | **Well-being** | | **Poor well-being** | |  |  | **No/mild distress** | | **Severe distress** | |  |  |  |
|  | N | % | N | % | Total | p | N | % | N | % | Total | p |  |
| **Overall** | 10,367 | 84.46 | 1,142 | 15.50 | 1,908 |  | 11,681 | 93.4 | 826 | 6.6 | 12,507 |  |  |
| **Age** |  |  |  |  |  | <.001 |  |  |  |  |  | <.001 |  |
| <55 years | 72 | 81.8 | 16 | 18.2 | 88 |  | 74 | 84.1 | 14 | 15.9 | 88 |  |  |
| 55-64 years | 986 | 77.9 | 280 | 22.1 | 1,266 |  | 1,115 | 87.2 | 163 | 12.8 | 1,278 |  |  |
| 65-74 years | 4,730 | 86.8 | 718 | 13.2 | 5,448 |  | 5,195 | 93.8 | 341 | 6.2 | 5,536 |  |  |
| 75-84 years | 3,987 | 84.5 | 732 | 15.5 | 4,719 |  | 4,581 | 94.9 | 246 | 5.1 | 4,827 |  |  |
| 85+ years | 592 | 78.5 | 162 | 21.5 | 754 |  | 716 | 92.0 | 62 | 8.0 | 778 |  |  |
| **Number of LTCs** |  |  |  |  |  | <.001 |  |  |  |  |  | <.001 |  |
| 0 | 2,651 | 88.8 | 336 | 11.2 | 2,987 |  | 2,938 | 96.5 | 108 | 3.5 | 3,046 |  |  |
| 1 | 3,741 | 87.5 | 534 | 12.5 | 4,275 |  | 4,179 | 95.7 | 188 | 4.3 | 4,367 |  |  |
| 2 | 2,233 | 82.8 | 464 | 17.2 | 2,697 |  | 2,542 | 92.7 | 199 | 7.3 | 2,741 |  |  |
| 3 | 1,019 | 77.5 | 296 | 22.5 | 1,315 |  | 1,198 | 89.3 | 144 | 10.7 | 1,342 |  |  |
| 4+ | 723 | 72.2 | 278 | 27.8 | 1,001 |  | 824 | 81.5 | 187 | 18.5 | 1,011 |  |  |
| **Employment** |  |  |  |  |  | <.001 |  |  |  |  |  | <.001 |  |
| Employed | 1,540 | 1,444 | 221 | 13.3 | 1,665 |  | 1,604 | 95.1 | 83 | 4.9 | 1,687 |  |  |
| Unemployed | 174 | 143 | 119 | 45.4 | 262 |  | 180 | 67.4 | 87 | 32.6 | 267 |  |  |
| Retired | 9,108 | 8,504 | 1,499 | 15.0 | 10,003 |  | 9,574 | 93.9 | 627 | 6.1 | 10,201 |  |  |
| Other | 44 | 34 | 16 | 32.0 | 50 |  | 44 | 89.8 | 5 | 10.2 | 49 |  |  |
| **Ethnicity** |  |  |  |  |  | 0.37 |  |  |  |  |  | 0.02 |  |
| White | 9,947 | 84.6 | 1,809 | 15.4 | 11,756 |  | 11,200 | 93.5 | 776 | 6.5 | 11,976 |  |  |
| Non-white | 198 | 82.5 | 42 | 17.5 | 240 |  | 223 | 89.9 | 25 | 10.1 | 248 |  |  |
| **Marital status** |  |  |  |  |  | <.001 |  |  |  |  |  | <.001 |  |
| Married/Civil Partner | 8,366 | 85.8 | 1,386 | 14.2 | 9,752 |  | 9,316 | 94.0 | 598 | 6.0 | 9,914 |  |  |
| Separated/Divorced | 640 | 78.1 | 179 | 21.9 | 819 |  | 748 | 88.4 | 98 | 11.6 | 846 |  |  |
| Widowed | 828 | 79.5 | 213 | 20.5 | 1,041 |  | 981 | 92.8 | 76 | 7.2 | 1,057 |  |  |
| Single | 346 | 80.1 | 86 | 19.9 | 432 |  | 419 | 92.9 | 32 | 7.1 | 451 |  |  |
| Other | 113 | 80.7 | 27 | 19.3 | 140 |  | 133 | 90.5 | 14 | 9.5 | 147 |  |  |
| **Deprivation quintile** |  |  |  |  |  | <.001 |  |  |  |  |  | <.001 |  |
| least deprived | 2,867 | 87.7 | 403 | 12.3 | 3,270 |  | 3,186 | 95.8 | 141 | 4.2 | 3,327 |  |  |
| 2 | 2,801 | 85.8 | 465 | 14.2 | 3,266 |  | 3,155 | 94.8 | 174 | 5.2 | 3,329 |  |  |
| 3 | 2,222 | 85.0 | 392 | 15.0 | 2,614 |  | 2,488 | 93.5 | 173 | 6.5 | 2,661 |  |  |
| 4 | 1,414 | 81.0 | 331 | 19.0 | 1,745 |  | 1,609 | 90.3 | 172 | 9.7 | 1,781 |  |  |
| most deprived | 822 | 74.8 | 277 | 25.2 | 1,099 |  | 972 | 86.9 | 147 | 13.1 | 1,119 |  |  |
| **Carer status** |  |  |  |  |  | 0.08 |  |  |  |  |  | 0.19 |  |
| No | 7,627 | 84.8 | 1,372 | 15.2 | 8,999 |  | 8,569 | 93.6 | 584 | 6.4 | 9,153 |  |  |
| Yes | 2,440 | 84.1 | 462 | 15.9 | 2,902 |  | 2,751 | 92.9 | 209 | 7.1 | 2,960 |  |  |
| **Mental Health help seeking** |  |  |  |  |  | <.001 |  |  |  |  |  | <.001 |  |
| No | 8,706 | 86.9 | 1,318 | 13.1 | 10,024 |  | 9,735 | 95.3 | 481 | 4.7 | 10,216 |  |  |
| Yes | 1,467 | 73.4 | 531 | 26.6 | 1,998 |  | 1,711 | 84.3 | 319 | 15.7 | 2,030 |  |  |
| **Stage** |  |  |  |  |  | 0.07 |  |  |  |  |  | 0.06 |  |
| I/II | 4,224 | 85.3 | 727 | 14.7 | 4,951 |  | 4,739 | 94.0 | 304 | 6.0 | 5,043 |  |  |
| III | 2,743 | 84.7 | 495 | 15.3 | 3,238 |  | 3,071 | 93.3 | 221 | 6.7 | 3,292 |  |  |
| IV | 2,110 | 82.7 | 440 | 17.3 | 2,550 |  | 2,404 | 92.6 | 193 | 7.4 | 2,597 |  |  |
| **BMI** |  |  |  |  |  | <.001 |  |  |  |  |  | <.001 |  |
| <25 (Under/healthy weight) | 2,702 | 84.9 | 481 | 15.1 | 3,183 |  | 3,052 | 94.3 | 185 | 5.7 | 3,237 |  |  |
| 25-30 (Overweight) | 4,738 | 87.1 | 704 | 12.9 | 5,442 |  | 5,243 | 94.8 | 288 | 5.2 | 5,531 |  |  |
| 30 + (Obese) | 2,235 | 80.9 | 529 | 19.1 | 2,764 |  | 2,564 | 90.7 | 262 | 9.3 | 2,826 |  |  |
| **Nation** |  |  |  |  |  | 0.47 |  |  |  |  |  | <.001 |  |
| England | 8,852 | 84.8 | 1,583 | 15.2 | 10,435 |  | 9,946 | 93.7 | 664 | 6.3 | 10,610 |  |  |
| Wales | 718 | 82.3 | 154 | 17.7 | 872 |  | 832 | 91.5 | 77 | 8.5 | 909 |  |  |
| Scotland | 506 | 81.5 | 115 | 18.5 | 621 |  | 584 | 91.4 | 55 | 8.6 | 639 |  |  |
| NI | 291 | 83.9 | 56 | 16.1 | 347 |  | 319 | 91.4 | 30 | 8.6 | 349 |  |  |

^a^p values represent level of significance in a chi-squared test, significance was indicated at the .05 level. LTC: Long-term conditions

**Online resource 5.** Complete case analysis. Multivariable associations between cancer-related symptoms, HRQL and psychological distress and mental well-being, when controlling for variables in the core models. Odds ratios (OR) and 95% confidence intervals (CI)

|  | **Poor mental well-being^b^** | | **Severe psychological distress** | |  |  |  |
| --- | --- | --- | --- | --- | --- | --- | --- |
| **Symptoms and HRQL** | N | OR (95% CI) | N | OR (95% CI) |  |  |  |
| **Cancer-related symptoms** |  |  |  |  |  |  |  |
| **Urinary bother** | **9,355** |  | **9,666** |  |  |  |  |
| No bother^r^ |  | 1.00 |  | 1.00 |  |  |  |
| Moderate/big bother |  | 2.92 (2.52-3.38) |  | 3.47 (2.86-4.20) |  |  |  |
| **Bowel bother** | **9,384** |  | **9,698** |  |  |  |  |
| No bother |  | 1.00 |  | 1.00 |  |  |  |
| Moderate/big bother |  | 2.28 (1.95-2.67) |  | 3.46 (2.83-4.23) |  |  |  |
| **Sexual bother** | **8,787** |  | **9,049** |  |  |  |  |
| No bother |  | 1.00 |  | 1.00 |  |  |  |
| Moderate/big bother |  | 1.84 (1.63-2.09) |  | 2.61 (2.15-3.18) |  |  |  |
| **Fatigue** | **8,822** |  | **9,082** |  |  |  |  |
| No fatigue |  | 1.00 |  | 1.00 |  |  |  |
| Fatigue |  | 3.86 (3.38-4.42) |  | 9.92 (7.63-12.89) |  |  |  |
| **Mobility** | **9,358** |  | **9,668** |  |  |  |  |
| No problems |  | 1.00 |  | 1.00 |  |  |  |
| Some problems |  | 2.81 (2.46-3.21) |  | 4.51 (3.61-5.63) |  |  |  |
| **Self-care** | **9,380** |  | **9,691** |  |  |  |  |
| No problems |  | 1.00 |  | 1.00 |  |  |  |
| Some problems |  | 3.89 (3.39-4.48) |  | 6.31 (5.20-7.66) |  |  |  |
| **Usual activities** | **9,356** |  | **9,668** |  |  |  |  |
| No problems |  | 1.00 |  | 1.00 |  |  |  |
| Some problems |  | 3.37 (2.94-3.86) |  | 6.31 (5.20-7.66) |  |  |  |
| **Pain/discomfort** | **9,344** |  | **9,655** |  |  |  |  |
| No problems |  | 1.00 |  | 1.00 |  |  |  |
| Some problems |  | 2.36 (2.08-2.69) |  | 3.39 (2.72-4.21) |  |  |  |
| **SAH** | **9910** | 0.96 (0.95-0.96) | **9,614** | 0.94 (0.94-0.95) |  |  |  |

SAH Self assessed health. This was a continuous variable scored out of a total of 100 where a greater score indicates better HRQL

**Online resource 6.** Comparison of age and stage by ADT treatment type. Numbers and percentages

|  | **Age** | | | | | | | | | | **Stage** | | | | | |
| --- | --- | --- | --- | --- | --- | --- | --- | --- | --- | --- | --- | --- | --- | --- | --- | --- |
|  | <55 years | | 55-64 years | | 65-74 years | | 75-84 years | | 85+ years | | I/II | | III | | IV | |
| **ADT type** | N | % | N | % | N | % | N | % | N | % | N | % | N | % | N | % |
| ADT alone | 5 | 0.2 | 129 | 4.1 | 803 | 25.8 | 1,538 | 49.4 | 640 | 20.6 | 965 | 37.6 | 487 | 19.0 | 1,116 | 43.5 |
| EBRT & ADT | 49 | 0.7 | 780 | 10.4 | 3,730 | 49.8 | 2,845 | 38.0 | 83 | 1.1 | 3,688 | 55.0 | 2,359 | 35.2 | 658 | 9.8 |
| ADT & EBRT & surgery | 18 | 2.0 | 177 | 19.6 | 447 | 49.6 | 250 | 27.8 | 9 | 1.0 | 311 | 39.8 | 349 | 44.7 | 121 | 15.5 |
| Surgery & ADT | 5 | 0.9 | 62 | 10.7 | 205 | 35.3 | 226 | 38.9 | 83 | 14.3 | 185 | 38.8 | 116 | 24.3 | 176 | 36.9 |
| ADT & systemic | 5 | 0.8 | 83 | 13.2 | 319 | 50.7 | 171 | 27.2 | 51 | 8.1 | 71 | 12.7 | 37 | 6.6 | 450 | 80.7 |
| ADT & EBRT & systemic | 7 | 1.8 | 78 | 20.5 | 192 | 50.4 | 98 | 25.7 | 6 | 1.6 | 56 | 16.4 | 93 | 27.3 | 192 | 56.3 |

ADT: Androgen Deprivation Therapy; EBRT: External Beam Radiotherapy

**Online resource 7.** Comparison of cancer-related symptoms in men with and without ADT. Numbers and percentages

|  |  | **ADT** | | **No ADT** | |  |
| --- | --- | --- | --- | --- | --- | --- |
| **Cancer related symptoms** | | **N** | **%** | **N** | **%** | **p** |
| **Age group** | <55 years | 89 | 0.7 | 500 | 2.9 |  |
|  | 55-64 years | 1,309 | 10.0 | 3,562 | 20.9 | <.001 |
|  | 65-74 years | 5,696 | 43.5 | 8,523 | 50.1 |  |
|  | 75-84 years | 5,128 | 39.2 | 3,858 | 22.7 |  |
|  | 85+ years | 872 | 6.7 | 572 | 3.4 |  |
| **Number of LTCs** | 0 | 3,192 | 24.4 | 5,615 | 33.0 |  |
|  | 1 | 4,551 | 34.8 | 6,040 | 35.5 | <.001 |
|  | 2 | 2,867 | 21.9 | 3,096 | 18.2 |  |
|  | 3 | 1,413 | 10.8 | 1,242 | 7.3 |  |
|  | 4+ | 1,074 | 8.2 | 1,021 | 6.0 |  |
| **Stage** | I/II | 5,276 | 46.2 | 11,450 | 79.0 |  |
|  | III | 3,441 | 30.1 | 2,511 | 17.3 | <.001 |
|  | IV | 2,713 | 23.7 | 532 | 3.7 |  |
| **Mobility** | No problem | 7,280 | 56.1 | 12,296 | 73.0 |  |
|  | Some problems | 5,687 | 43.9 | 4,542 | 27.0 | <.001 |
| **Self-care** | No problem | 10,707 | 82.4 | 15,193 | 90.1 |  |
|  | Some problems | 2,289 | 18.0 | 1,661 | 9.9 | <.001 |
| **Usual activities** | No problem | 6,990 | 53.9 | 11,958 | 71.0 |  |
|  | Some problems | 5,980 | 46.1 | 4,886 | 29.0 | <.001 |
| **Pain/discomfort** | No problem | 6,740 | 52.1 | 10,841 | 64.5 |  |
|  | Some problems | 6,203 | 47.9 | 5,969 | 35.5 | <.001 |
| **Urinary bother** | No bother | 11,193 | 86.5 | 14,787 | 87.9 |  |
|  | Moderate/big bother | 1,744 | 13.5 | 2,040 | 12.1 | <.001 |
| **Bowel bother** | No bother | 11,499 | 88.5 | 15,786 | 93.7 |  |
|  | Moderate/big bother | 1,491 | 11.5 | 1,068 | 6.3 | <.001 |
| **Sexual bother** | No bother | 6,541 | 54.7 | 8,873 | 55.8 |  |
|  | Moderate/big bother | 5,426 | 45.3 | 7,033 | 44.2 | 0.06 |
| **Fatigue** | No fatigue | 7,353 | 61.4 | 12,158 | 79.0 |  |
|  | Fatigue | 4,619 | 38.6 | 3,230 | 21.0 | <.001 |
| **SAH (mean, SD)** |  | 74 | 18.6 | 79.5 | 16.5 | <.001 |
| **SWEMWBS** | Wellbeing | 10,367 | 84.5 | 13,799 | 86.5 | <.001 |
|  | Poor wellbeing | 1,908 | 15.5 | 2,157 | 13.5 |  |
| **K6** | No distress | 11,681 | 93.4 | 15,418 | 95.2 |  |
|  | Distress | 826 | 6.6 | 777 | 4.8 | <.001 |

LTC: Long-term conditions; VAS EQ-5D Visual Analogue Scale; SAH Self assessed health. This was a continuous variable scored out of a total of 100 where a greater score indicates better HRQL

P values of significance based on Chi^2^ test.
